# Traumatic complete pyloric transection following blunt abdominal trauma: A case report and literature review

**DOI:** 10.1097/MD.0000000000050339

**Published:** 2026-08-21

**Authors:** Khac Thao Thai, Trong Hoe Nguyen, Thanh Son Le, Van Dan Nguyen, Doanh Hieu Tran

**Affiliations:** a Department of Gastrointestinal Surgery, Military Hospital 103, Hanoi, Vietnam; b Department of Hepatobiliary and Pancreatic Surgery, Military Hospital 103, Hanoi, Vietnam; c Department of Diagnostic Radiology, Military Hospital 103, Hanoi, Vietnam.

**Keywords:** blunt abdominal trauma, computed tomography, emergency surgery, gastric rupture, pyloric transection

## Abstract

**Rationale:**

Gastric rupture after blunt abdominal trauma is rare, and complex disruptions such as complete pyloric transection are exceptionally uncommon, having been described only in isolated case reports. Early diagnosis and timely surgical intervention are crucial to prevent septic complications resulting from peritoneal contamination.

**Patient concerns:**

A 31-year-old male presented with severe abdominal pain after a motorcycle collision following a heavy meal and alcohol intake.

**Diagnoses:**

The patient presented with agitation and impaired communication due to alcohol intoxication. Vital signs included a respiratory rate of 28 breaths/min, a pulse of 112 bpm, blood pressure of 110/60 mm Hg, and he was afebrile. Abdominal examination revealed epigastric bruising, mild distension, diffuse tenderness with epigastric predominance, abdominal guarding, and rebound tenderness suggestive of peritonitis. Whole-body contrast-enhanced computed tomography demonstrated pneumoperitoneum, extraluminal food spillage, and a clear discontinuity of the gastric wall, consistent with traumatic gastric rupture.

**Interventions:**

Emergency laparoscopy converted to laparotomy revealed complete pyloric transection, anterior antral rupture, and colonic seromuscular tears. The patient underwent two-thirds distal gastrectomy with Roux-en-Y reconstruction, extensive peritoneal lavage, and abdominal drainage.

**Outcomes:**

The postoperative course was favorable. Oral intake resumed on postoperative day 5, and the patient was discharged in stable condition on day 20. At the 30-day follow-up, he had normal eating and bowel habits, gained 3 kg, and endoscopy demonstrated a healed and patent anastomosis.

**Lessons:**

Whole-body contrast-enhanced computed tomography is highly valuable in the early evaluation of blunt polytrauma and may replace routine radiographs in centers with rapid pan-scan capability. However, when computed tomography is negative or inconclusive, operative decisions should still rely on clinical deterioration such as progressive abdominal distension and guarding. Complex gastric injuries, including complete pyloric transection, require prompt resection and restoration of gastrointestinal continuity to achieve favorable outcomes.

## 1. Introduction

The incidence of gastric injury after blunt abdominal trauma ranges from 0.02% to 2.8%,^[1–3]^ whereas gastric rupture is far less common, occurring in up to 1.2% of cases.^[2–5]^ Most published cases involve simple full-thickness perforations, and a complete transection of the stomach itself appears exceptionally rare, with only a few isolated cases of traumatic pyloric or gastroduodenal transection reported in the literature. Despite its rarity, traumatic gastric rupture may result in substantial morbidity and mortality, particularly in the presence of delayed diagnosis or associated injuries. Shinkawa et al^[2]^ reported associated injuries in 93% of patients and an overall mortality rate of 14%, with deaths mainly related to massive hemorrhage, sepsis, and multiple organ failure. Similarly, Rodríguez-Hermosa et al^[3]^ observed postoperative complications in 60% of patients and a mortality rate of 4%, emphasizing the potentially life-threatening nature of these injuries and the importance of early diagnosis and prompt surgical intervention.

Among the complex gastric injuries reported in the literature, Kimmins et al^[6]^ described a rare case of near-complete gastric transection, whereas Herath et al^[7]^ and Vidit et al^[8]^ reported complete transections at the pyloric or gastroduodenal junction. Although favorable outcomes have generally been achieved following early diagnosis and timely surgical intervention, delayed gastric dysfunction secondary to traumatic vagal injury was reported by Kimmins et al.^[6]^

We present a rare case of complex traumatic gastric rupture with complete pyloric transection following motor vehicle trauma, highlighting the diagnostic contribution of whole-body computed tomography (WBCT) and discussing operative management supported by current international literature.

## 2. Case presentation

A 31-year-old previously healthy man was brought to the emergency department 1.5 hours after a motorcycle crash. He reported striking the epigastrium against the concrete edge of a roadside canal. He remained conscious throughout the event and recalled the immediate onset of severe upper abdominal pain. He had eaten a large meal and consumed several beers approximately 1 hour before the accident.

On arrival, the patient was markedly agitated and difficult to communicate with because of alcohol intoxication and severe abdominal pain. His Glasgow Coma Scale (GCS) score was E4V3M4 (Eye opening 4, Verbal response 3, Motor response 4), and his blood ethanol concentration was 219.51 mg/dL. Oxygen supplementation at 6 L/min by mask was provided. Vital signs showed a respiratory rate of 28 breaths/min, pulse 112 bpm, blood pressure 110/60 mm Hg, and an afebrile temperature. Multiple superficial abrasions were present on the upper and lower extremities. A prominent contusion was noted over the epigastrium. The abdomen was mildly distended with diffuse tenderness, most pronounced in the epigastric region, accompanied by guarding and rebound tenderness, indicating peritoneal irritation.

Laboratory investigations demonstrated leukocytosis (white blood cell count: 14.9 × 10^9^/L) with neutrophilia (absolute neutrophil count: 10.2 × 109/L). Arterial blood gas analysis revealed metabolic acidosis (pH: 7.247, bicarbonate: 16 mmol/L, base excess: −9.8 mmol/L) and elevated serum lactate (4.8 mmol/L), indicating significant physiological stress and intra-abdominal contamination.

Focused assessment with sonography for trauma (FAST) was performed in the emergency department and showed no evidence of free intraperitoneal fluid. Despite the negative FAST findings, the patient remained hemodynamically stable but exhibited clear signs of generalized peritonitis after high-energy blunt abdominal trauma. Therefore, WBCT was subsequently performed to accurately identify the injured organ, evaluate associated injuries, and facilitate operative planning.

**Figure 1.**
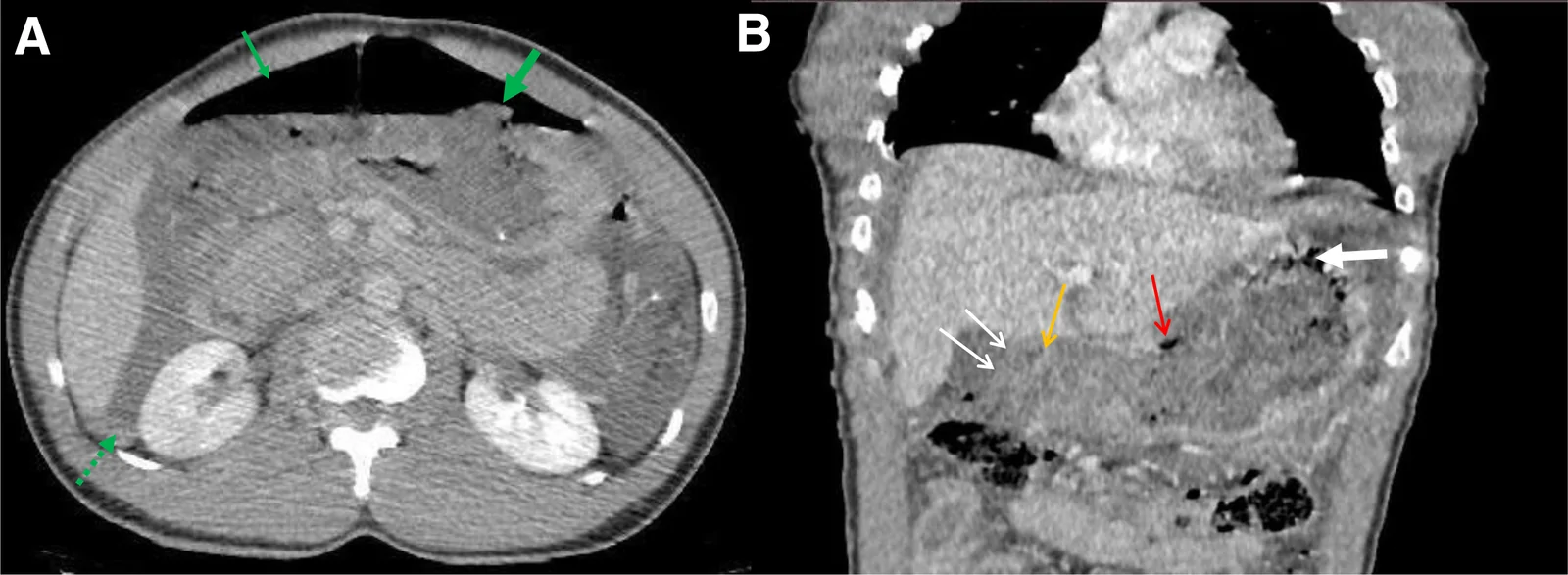
CT features of blunt traumatic gastric rupture. (A) Axial contrast-enhanced CT demonstrates a large volume of intraperitoneal free air accumulating anterior to the stomach (green arrow), extraluminal fluid leaking through the gastric perforation into the peritoneal cavity (large green arrow), and free fluid in the Morrison pouch (green dashed line). (B) Coronal reconstruction shows an irregular full-thickness rupture along the anterior gastric antrum with associated transmural air (large white arrow), an irregular heterogeneous soft-tissue density at the pyloroduodenal junction, suggestive of severe soft-tissue disruption (small white arrow), a hyperattenuating mucosal layer along the posterior wall of the pylorus (yellow arrow), and subhepatic free air (red arrow). CT = computed tomography.

Imaging demonstrated a large amount of free intraperitoneal air, extraluminal food material predominantly beneath the liver, and focal discontinuity of the anterior gastric wall, highly suggestive of gastric rupture. No solid organ injury was identified (Fig. 1).

**Figure 2.**
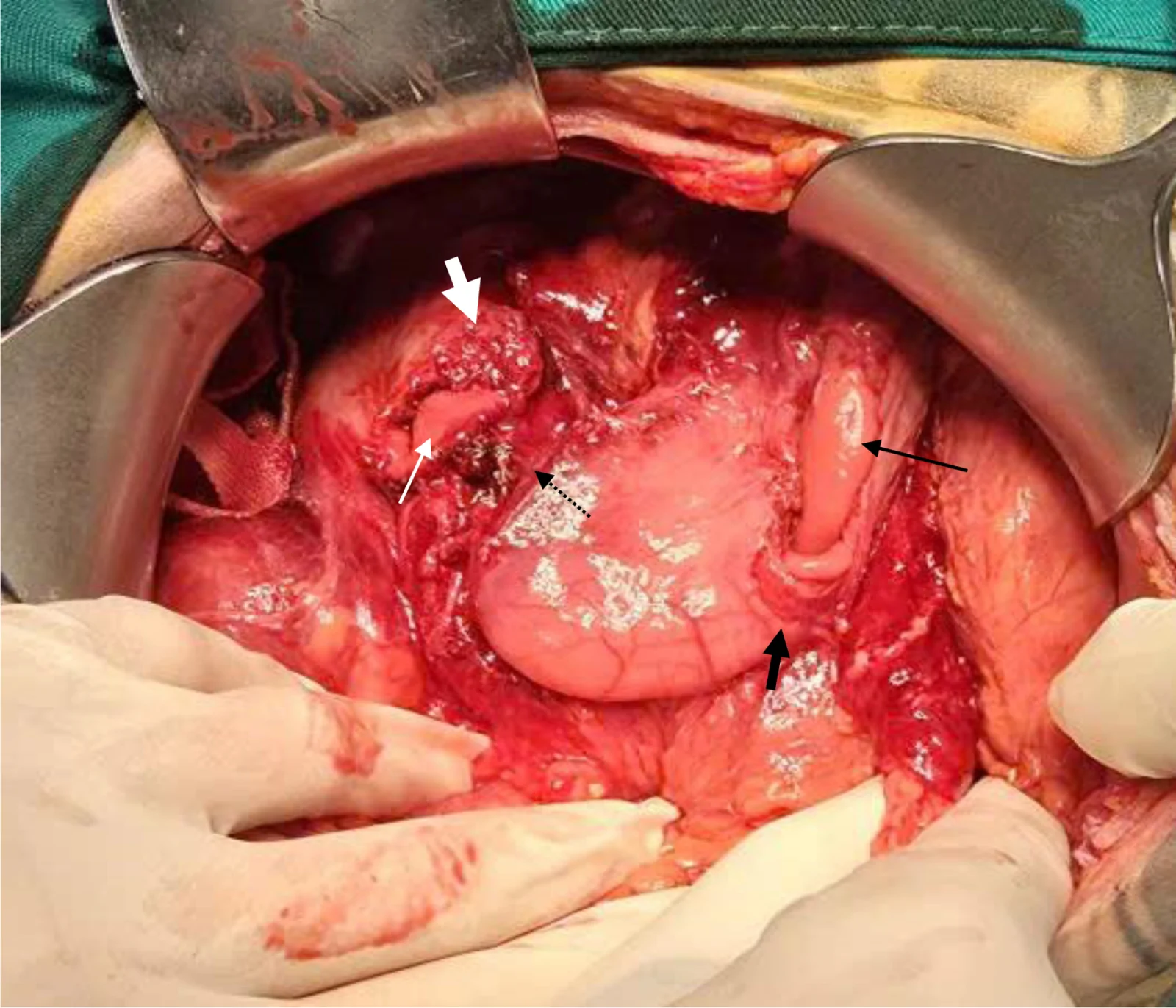
Intraoperative findings of the pyloric transection and complex gastric injuries. The operative field demonstrates the contused transected pyloric stump (large white arrow) and the exposed posterior pyloric mucosa (small white arrow). A separate rupture line is visible along the gastric wall (black dashed arrow), together with an irregular full-thickness anterior antral tear (large black arrow) and exposed posterior gastric mucosa (small black arrow).

The patient was intubated for airway protection, placed on mechanical ventilation, and received analgesia, sedation, fluid resuscitation, electrolyte correction, and broad-spectrum antibiotics. An emergency operation was indicated. A diagnostic laparoscopy was first performed using 3 trocars. The peritoneal cavity contained a large volume of food material and digestive fluid, and the gastric injury appeared highly complex. Given the extent of contamination and the inability to manage the injuries laparoscopically, the procedure was converted to an open laparotomy through a 20-cm upper midline incision. Upon entering the abdomen, all food debris and digestive fluid were evacuated, followed by thorough warm-saline irrigation. Detailed exploration revealed a complete transection of the pylorus, accompanied by a separate full-thickness rupture of the anterior gastric antrum approximately 4 cm proximal to the transection site. Multiple contusions and seromuscular tears were present along the gastric wall. Examination of other intra-abdominal organs showed 3 seromuscular tears on the transverse colon, each measuring 1–3 cm in length, with no additional injuries identified. According to the American Association for the Surgery of Trauma Organ Injury Scale, the gastric injury was classified as grade V (tissue loss or devascularization involving ≥ 50% of the stomach)^[9]^ (Fig. 2).

A two-thirds distal gastrectomy encompassing all devitalized gastric tissue and the disrupted pyloric segment was performed. After resection of the injured pyloric segment and confirmation of viable tissue margins, the duodenal stump was closed at the healthy first portion of the duodenum (D1) in a fashion similar to routine distal gastrectomy. Reconstruction was subsequently achieved using a Roux-en-Y gastrojejunostomy. Seromuscular tears of the transverse colon were repaired primarily. The peritoneal cavity was thoroughly irrigated with warm saline, and abdominal drains were placed. The patient was transferred to the intensive care unit postoperatively.

During the immediate postoperative period, he developed transient tachycardia and borderline hypotension. Given the severity of the abdominal trauma, major gastric resection with reconstruction, and perioperative blood loss, supportive transfusion therapy consisting of 2 units of packed red blood cells and 2 units of fresh frozen plasma was administered. His hemodynamic status subsequently stabilized, and no additional transfusion or reoperation was required.

The patient required transient vasopressor support immediately after surgery but was successfully weaned within the first postoperative day. He was extubated on postoperative day 1 and subsequently remained hemodynamically stable. Bowel function gradually recovered, with passage of flatus on postoperative day 4 and initiation of oral intake on postoperative day 5.

A superficial incisional surgical-site infection developed on postoperative day 4, manifested by wound exudation and requiring local wound opening, wound care, and antibiotic therapy. According to the Clavien-Dindo classification, this represented a grade II postoperative complication. The patient showed progressive clinical improvement thereafter, with normal bowel function and adequate oral intake. Abdominal drains were removed on postoperative day 7, and he was discharged in stable condition on postoperative day 20.

**Figure 3.**
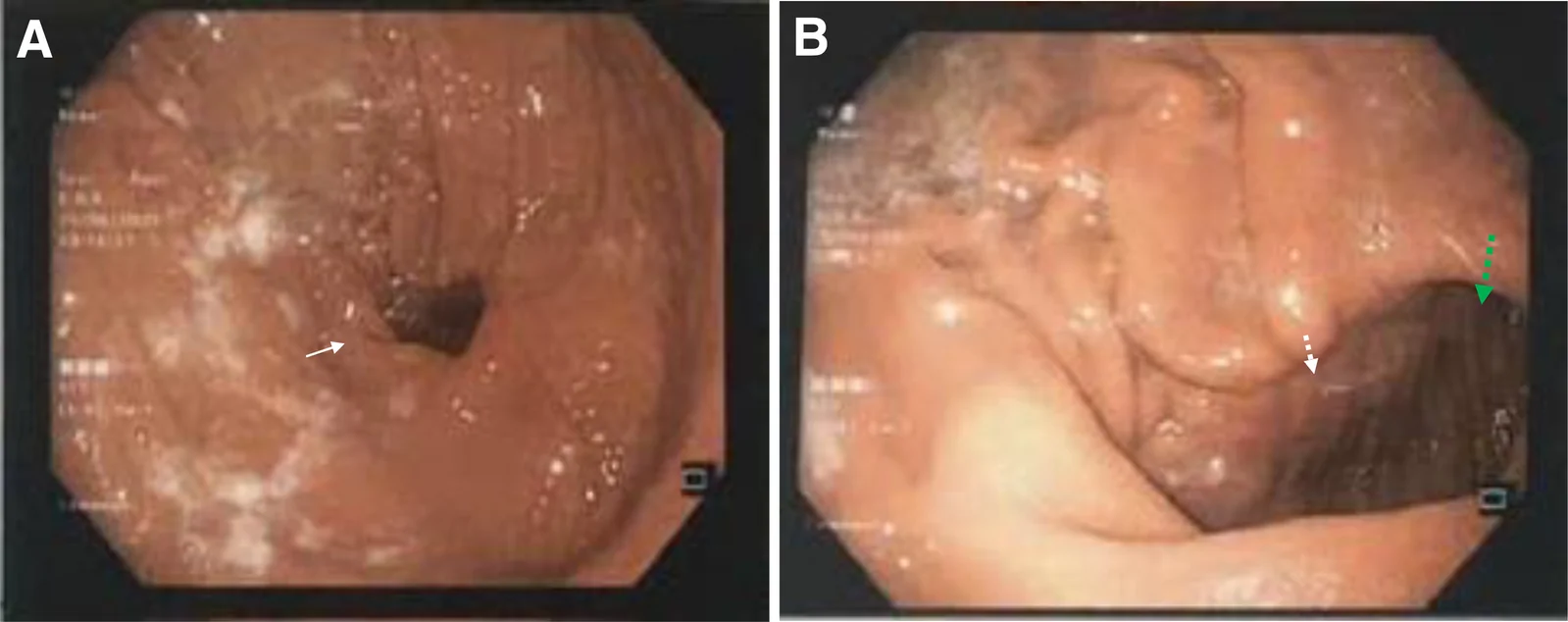
Endoscopic evaluation 30 days after hospital discharge. (A) Endoscopic view showing normal gastric mucosa surrounding a well-healed, pinkish gastrojejunal anastomosis (white arrow). (B) Widely patent anastomotic lumen (green dashed arrow) and a smooth, well-defined anastomotic line (white dashed arrow).

At the 30-day follow-up visit, the patient had gained approximately 3 kg, tolerated a normal diet, reported normal bowel habits without abdominal pain, nausea, or vomiting, and had resumed normal daily activities. He expressed satisfaction with the treatment outcome. Upper gastrointestinal endoscopy demonstrated a well-healed and patent gastrojejunal anastomosis without evidence of leakage or stricture (Fig. 3).

This case report was prepared in accordance with the CARE guidelines. The use and publication of the clinical data and accompanying images were authorized by Military Hospital 103, Hanoi, Vietnam. Written informed consent for publication was obtained from the patient, and all identifying information was anonymized before manuscript preparation.

## 3. Discussion

Traumatic gastric rupture is an uncommon consequence of blunt abdominal trauma, representing only 0.02% to 2.8% of all cases, with true full-thickness disruption occurring in fewer than 1.2% of patients.^[1–5]^ The rarity of this injury reflects the protective characteristics of the stomach, including its thick muscular wall, rich vascularity, mobility, and shielding by surrounding organs.

The injury pattern in this case is most consistent with a combined mechanism in which acute intragastric overpressure and high-energy shear–compression acted simultaneously. The large, irregular antral rupture reflects a burst-type injury produced when a distended stomach is abruptly compressed.^[10]^ In contrast, shear–compression forces generated during high-velocity deceleration can produce deeper disruptions, including vertical transection across the pylorus or gastroduodenal junction.^[2,11,12]^ Similar high-shear injuries have been described in reports of gastroduodenal transection requiring substantial deceleration forces across the pylorus–duodenum junction.^[7,8]^ The coexistence of 2 morphologically distinct lesions strongly supports the involvement of both mechanisms in this patient.

Clinical manifestations of traumatic gastric rupture are often nonspecific, especially when intoxication, altered mental status, or competing injuries obscure abdominal findings. Rodríguez-Hermosa et al^[3]^ demonstrated that delayed diagnosis is associated with increased morbidity and mortality, emphasizing the importance of maintaining a high index of suspicion in patients sustaining high-energy blunt abdominal trauma. Likewise, Fakhry et al^[13]^ reported that even relatively short delays in operative intervention significantly increase morbidity in hollow-viscus injuries.

Although plain radiography and FAST remain useful screening modalities, both have limited sensitivity for gastric injuries because they frequently fail to identify free intraperitoneal air, food debris, or focal gastric-wall defects.^[14,15]^ Consequently, WBCT has become the preferred imaging modality in stable or borderline-stable trauma patients.^[16]^ Consistent with previous reports, WBCT in the present case accurately identified imaging features highly suggestive of hollow-viscus injury, allowing early operative planning before the development of further physiological deterioration. Compared with plain radiography and FAST, computed tomography also delineated the extent of gastric disruption and associated intra-abdominal contamination.

The injury severity and immediate concern for hollow-viscus disruption mandated urgent operative management. Diagnostic laparoscopy was used initially because modern evidence demonstrates that it is safe, accurate, and reduces surgical trauma in selected trauma patients.^[17]^ Unlike previously reported cases of pyloric or gastroduodenal transection that underwent immediate exploratory laparotomy,^[6–8]^ the present case illustrates that an initial diagnostic laparoscopy can provide valuable intraoperative assessment without delaying definitive open management when conversion criteria are promptly recognized.

However, guidelines consistently advise conversion to open surgery when gross contamination, unclear anatomy, or complex gastrointestinal injuries are encountered.^[18]^ As demonstrated in this patient, the laparoscopic findings fulfilled these criteria, justifying prompt conversion to laparotomy. Once adequate exposure had been achieved, operative management was guided by the extent of tissue destruction and viability rather than by anatomical disruption alone. While simple perforations without ischemia are amenable to primary closure, destructive injuries featuring devascularization, segmental loss, or complete transection require resection – an approach consistent with World Society of Emergency Surgery bowel injury guidelines recommending resection when more than 50% of the wall is compromised, or mesenteric perfusion is impaired.^[19]^ Accordingly, definitive resection with Roux-en-Y reconstruction was selected to achieve complete removal of devitalized tissue, secure well-vascularized margins, and minimize tension on the reconstruction.^[20]^ Associated colonic injuries were repaired primarily, and thorough peritoneal lavage was performed to reduce the risk of postoperative infectious complications.

Favorable recovery in this patient likely reflects early diagnosis, prompt contamination control, and definitive reconstruction before irreversible physiologic deterioration occurred. Previous reports of pyloric or gastroduodenal transection have generally demonstrated favorable outcomes when early diagnosis and timely surgical intervention were achieved (Table 1). Kimmins et al^[6]^ described an 11-year-old boy with near-complete distal gastric transection following a seat-belt injury who underwent primary repair but subsequently developed delayed gastric dysfunction attributed to traumatic vagal injury. Herath et al^[7]^ reported complete gastroduodenal transection associated with multiple concomitant injuries and successfully managed the patient using a staged approach with Roux-en-Y reconstruction, resulting in excellent functional recovery. Likewise, Vidit et al^[8]^ described successful management of complete gastroduodenal transection by primary repair combined with Witzel feeding jejunostomy, without major postoperative complications.

**Table 1:** Previously reported cases of pyloric/gastroduodenal transection.

Author	Year	Age/sex	Mechanism	Injury pattern	Associated injury	Management	Outcome
Kimmins et al ^[6]^	1996	11/Male	Seat-belt MVC	Near-complete distal gastric transection	Transverse colon serosal tears	Primary repair	Delayed traumatic vagotomy, eventual recovery
Herath et al ^[7]^	2020	35/Female	Improper seat-belt injury (80 km/h MVC)	Complete gastroduodenal junction transection	Splenic injury, transverse colon injury, left rib and humeral fractures	Damage-control surgery → delayed Roux-en-Y gastrojejunostomy	Good recovery
Vidit et al. ^[8]^	2023	18/Male	High-velocity road traffic accident	Complete gastroduodenal transection	None reported	Primary repair + Witzel feeding jejunostomy	Uneventful recovery, discharged POD10
Present case	2025	31/Male	Motorcycle crash against concrete canal edge	Complete pyloric transection + large anterior antral rupture + seromuscular colonic tears	Transverse colon injury	Distal gastrectomy + Roux-en-Y gastrojejunostomy	Superficial SSI only; excellent recovery

MVC = motor vehicle collision, POD = postoperative day, SSI = surgical-site infection.

Compared with these previously reported cases, our patient demonstrated a uniquely complex injury pattern characterized by simultaneous complete pyloric transection, a separate large full-thickness anterior antral rupture, diffuse food contamination, and associated colonic injuries. In addition, unlike previous reports that proceeded directly to exploratory laparotomy, our patient initially underwent diagnostic laparoscopy, which rapidly confirmed the severity of contamination and facilitated operative planning before conversion to open surgery. To our knowledge, reports describing an initial laparoscopic approach in such complex pyloric injuries remain limited.

Despite the greater anatomical complexity of the present case, excellent short-term outcomes were achieved through prompt diagnosis, early source control, extensive peritoneal lavage, and definitive reconstruction with distal gastrectomy and Roux-en-Y gastrojejunostomy.

Although immediate definitive reconstruction was feasible in this patient, damage control surgery should remain an important consideration in more physiologically compromised individuals. In the presence of profound shock, persistent metabolic acidosis, coagulopathy, hypothermia, or extensive associated injuries, abbreviated laparotomy with rapid hemorrhage and contamination control followed by staged reconstruction may improve survival by allowing physiologic resuscitation before definitive repair. The successful staged management reported by Herath et al^[7]^ further illustrates that operative strategy should be individualized according to the patient’s physiologic reserve rather than injury morphology alone. In our case, the absence of refractory shock, uncontrolled hemorrhage, or severe coagulopathy permitted safe immediate definitive reconstruction.

Several limitations should be acknowledged. This report represents a single case from a single institution and therefore cannot establish generalizable recommendations regarding the optimal reconstructive strategy for destructive gastric injuries. In addition, follow-up was relatively short, precluding assessment of long-term nutritional outcomes, gastric function, and quality of life after distal gastrectomy and Roux-en-Y reconstruction.

## 4. Conclusion

Traumatic gastric rupture with complete pyloric transection is exceedingly rare and may be overlooked when clinical findings are obscured by intoxication or associated injuries. In patients sustaining high-energy blunt abdominal trauma, WBCT plays a pivotal role in early diagnosis and operative planning. This case highlights that management should be individualized according to injury severity, tissue viability, associated injuries, and physiologic status. Early recognition, prompt source control, and individualized reconstruction can result in favorable outcomes even in complex multisite gastric injuries.

## Acknowledgments

The authors would like to thank the medical and nursing staff of Military Hospital 103 for their contribution to the management of this patient.

## Author contributions

**Conceptualization:** Khac Thao Thai, Trong Hoe Nguyen, Thanh Son Le, Doanh Hieu Tran.

**Data curation:** Khac Thao Thai, Trong Hoe Nguyen, Van Dan Nguyen, Doanh Hieu Tran.

**Investigation:** Khac Thao Thai, Trong Hoe Nguyen, Van Dan Nguyen, Doanh Hieu Tran.

**Methodology:** Khac Thao Thai, Trong Hoe Nguyen, Thanh Son Le, Doanh Hieu Tran.

**Supervision:** Trong Hoe Nguyen, Thanh Son Le.

**Writing – original draft:** Khac Thao Thai, Doanh Hieu Tran.

**Writing – review & editing:** Khac Thao Thai, Trong Hoe Nguyen, Thanh Son Le, Van Dan Nguyen, Doanh Hieu Tran.

## References

[1] Pontius GV Kilbourne BC Paul EG. Nonpenetrating abdominal trauma. AMA Arch Surg. 1956;72:800–11. 13312848 10.1001/archsurg.1956.01270230064008

[2] Shinkawa H Yasuhara H Naka S. Characteristic features of abdominal organ injuries associated with gastric rupture in blunt abdominal trauma. Am J Surg. 2004;187:394–7. 15006569 10.1016/j.amjsurg.2003.12.018

[3] Rodríguez-Hermosa JI Roig J Sirvent JM. Gastric perforations from abdominal trauma. Dig Surg. 2008;25:109–16. 18379189 10.1159/000121906

[4] Cox EF. Blunt abdominal trauma. A 5-year analysis of 870 patients requiring celiotomy. Ann Surg. 1984;199:467–74. 6712323 10.1097/00000658-198404000-00015 PMC1353367

[5] Tejerina Alvarez EE Holanda MS López-Espadas F Dominguez MJ Ots E Díaz-Regañón J. Gastric rupture from blunt abdominal trauma. Injury. 2004;35:228–31. 15124787 10.1016/s0020-1383(03)00212-2

[6] Kimmins MH Poenaru D Kamal I. Traumatic gastric transection: a case report. J Pediatr Surg. 1996;31:757–8. 8783094 10.1016/s0022-3468(96)90124-4

[7] Herath M Bautz P Parker D Dobbins C. The importance of wearing a seatbelt correctly – a case report of blunt trauma causing complete shearing transection of the gastroduodenal junction. Int J Surg Case Rep. 2020;72:197–201. 32544828 10.1016/j.ijscr.2020.05.008 PMC7298554

[8] Vidit Ranga HR Verma S Dushyant Y Mahipal Arora BK. Complete gastroduodenal transection in a blunt abdominal trauma patient: a rare case report. Int Surg J. 2023;10:2013–6.

[9] Moore EE Jurkovich GJ Knudson MM. Organ injury scaling. VI: extrahepatic biliary, esophagus, stomach, vulva, vagina, uterus (nonpregnant), uterus (pregnant), fallopian tube, and ovary. J Trauma. 1995;39:1069–70. 7500395 10.1097/00005373-199512000-00009

[10] Ueda S Okamoto N Seki T Matuyama T. A large gastric rupture due to blunt trauma: a case report and a review of the Japanese literature. J Surg Case Rep. 2021;2021:rjaa521. 33569160 10.1093/jscr/rjaa521 PMC7853660

[11] Shah H Sabbah BN Elwy BA Arabi TZ Sabbah AN Shah SY. Duodenal transection following a seat belt injury: a case report. Int J Surg Case Rep. 2022;96:107272. 35704986 10.1016/j.ijscr.2022.107272 PMC9198315

[12] Balasubramanian G Vijayakumar C Anbarasu I Sudharsanan S Raj Kumar N Baskaran D. Isolated rupture of duodenum following blunt trauma abdomen: report of a case of avulsion of pylorus. Int Surg J. 2017;4:2845–7.

[13] Fakhry SM Brownstein M Watts DD Baker CC Oller D. Relatively short diagnostic delays (<8 hours) produce morbidity and mortality in blunt small bowel injury: an analysis of time to operative intervention in 198 patients from a multicenter experience. J Trauma. 2000;48:408–14; discussion 414. 10744277 10.1097/00005373-200003000-00007

[14] Gans SL Stoker J Boermeester MA. Plain abdominal radiography in acute abdominal pain; past, present, and future. Int J Gen Med. 2012;5:525–33. 22807640 10.2147/IJGM.S17410 PMC3396109

[15] Stengel D Bauwens K Sehouli J. Emergency ultrasound-based algorithms for diagnosing blunt abdominal trauma. Cochrane Database Syst Rev. 2005:CD004446. 15846717 10.1002/14651858.CD004446.pub2

[16] Arruzza E Chau M Dizon J. Systematic review and meta-analysis of whole-body computed tomography compared to conventional radiological procedures of trauma patients. Eur J Radiol. 2020;129:109099. 32563164 10.1016/j.ejrad.2020.109099

[17] Wang J Cheng L Liu J. Laparoscopy vs. laparotomy for the management of abdominal trauma: a systematic review and meta-analysis. Front Surg. 2022;9:817134. 35350141 10.3389/fsurg.2022.817134 PMC8957831

[18] Fu JN Zhou L Ma T. The role of laparoscopy in closed abdominal injury. Heliyon. 2023;9:e20705. 37860552 10.1016/j.heliyon.2023.e20705 PMC10582471

[19] Smyth L Bendinelli C Lee N. WSES guidelines on blunt and penetrating bowel injury: diagnosis, investigations, and treatment. World J Emerg Surg. 2022;17:13. 35246190 10.1186/s13017-022-00418-y PMC8896237

[20] Stewart RM Livingston DH. Stomach and small bowel. In: Moore EE Mattox KL Feliciano DV, eds. Trauma. 9th ed. McGraw-Hill; 2020:707–8.

